# Effects of chlorantraniliprole on the life history traits of fall armyworm *Spodoptera frugiperda* (Lepidoptera: Noctuidae)

**DOI:** 10.3389/fphys.2023.1155455

**Published:** 2023-03-30

**Authors:** Ali Hasnain, Shuirong Zhang, Qinghua Chen, Lijuan Xia, Yutong Wu, Changwei Gong, Xuemei Liu, Pu Jian, Lei Zhang, Xuegui Wang

**Affiliations:** ^1^ State Key Laboratory of Crop Gene Exploration and Utilization in Southwest China, Sichuan Agricultural University, Chengdu, China; ^2^ College of Agriculture, Sichuan Agricultural University, Chengdu, China; ^3^ College of Plant Protection, Nanjing Agricultural University, Nanjing, China; ^4^ Key Laboratory of Integrated Pest Management on Crops in Southwest, Institute of Plant Protection, Sichuan Academy of Agricultural Sciences, Ministry of Agriculture, Chengdu, China; ^5^ Talent Development Service Center, Sichuan Provincial Department of Agriculture and Rural Affairs, Chengdu, China; ^6^ Department of Entomology, China Agricultural University, Beijing, China

**Keywords:** *Spodoptera frugiperda*, resistance, life history traits, chlorantraniliprole, vitellogenin

## Abstract

**Introduction:** *Spodoptera frugiperda* is an important nomadic agricultural pest with a diverse host range and resistance against several insecticides. The current study investigated the life history traits of two strains of the field-collected population against chlorantraniliprole using an age-stage two-sex life table.

**Method:** For this, we established the chlorantraniliprole-susceptible (Crp-SUS G_12_), and chlorantraniliprole-reduced susceptible (Crp-RES G_12_) strains derived from the sixth generation of the QJ-20 population having a resistance ratio (RR) of 10.39-fold, compared with the reported susceptible population.

**Results:** The results showed that the chlorantraniliprole-reduced susceptible strain attained a 4.0-fold RR, while the chlorantraniliprole-susceptible strain attained an RR of 0.85-fold, having overlapped fiducial limits (FLs) with the referred susceptible baseline. Meanwhile, the present study revealed that the development time of the susceptible strain was significantly longer than that of the reduced susceptible strain. Similarly, the mean longevity, adult pre-oviposition period (APOP), and total pre-oviposition period (TPOP) of the female chlorantraniliprole-susceptible strain were considerably longer than those of the female chlorantraniliprole-reduced susceptible strain. Contrarily, the population parameters, including the intrinsic rate of increase (*r*), finite rate of increase (*λ*), and net reproductive rate (*R*), of the chlorantraniliprole-susceptible strain were considerably lower than those of the chlorantraniliprole-reduced susceptible strain, while the mean generation time (*T*) of the chlorantraniliprole-susceptible strain was substantially longer than the chlorantraniliprole-reduced susceptible strain. The age-stage characteristic survival rate (*s*_*xj*_) and age-stage characteristic life expectancy (*e*_*xj*_) of the chlorantraniliprole-susceptible strain were longer than those of the chlorantraniliprole-reduced susceptible strain, but the age-stage-specific reproductive value (*v*_*xj*_) of the chlorantraniliprole-susceptible strain was shorter than that of the chlorantraniliprole-reduced susceptible strain. Moreover, the contents of vitellogenin (Vg) and VgR in the chlorantraniliprole-reduced susceptible strain were higher than those in the chlorantraniliprole-susceptible strain.

**Discussion:** These findings showed that reducing susceptibility to chlorantraniliprole promoted population growth in *S. frugiperda*. Therefore, this study could provide conceptual support for the integrated pest management (IPM) approach to control *S. frugiperda* in the field.

## 1 Introduction

The fall armyworm (FAW), *Spodoptera frugiperda* (Lepidoptera, Noctuidae), a devastative agricultural pest in its innate range, North and South America, and during the last 10 years, has become a significant invasive pest on a global scale. Numerous variables, including a high reproductive capacity, long-distance migration, and multiple host plants, have been implicated in the widespread FAW (Barros et al., 2010; Westbrook et al., 2016). Additionally, it may move from overwintering areas to other suitable climate zones without entering diapause (Sparks, 1979; Hardke et al., 2015). FAW was first reported in China in January 2019, and there are several reports on severe damage caused by FAW to the fields of maize, rice, and wheat, threatening the food supply and causing yield loss in China (Xiao, 2021; Zhang et al., 2021). The continuous application of insecticides causes resistance in the pest population over time (Denholm and Devine, 2013). For FAW, the resistance development has been reported for about 29 different insecticides from America by 2017 (Gutierrez-Moreno et al., 2019) and is attributed to attaining exceptional deviations in physiology, behavior, reproduction, longevity, and biology (Haynes, 1988; Teke and Mutlu, 2021; Liu et al., 2022). The life history parameters, including insect fertility, mortality, and lifespan, can be better studied by using the age-stage two-sex life table because it computes the data by considering both sexes in the calculations, as compared to the conventional life table, which only focuses on the female population (Abdel-Khalek and Momen, 2022; Younas et al., 2022). Many studies have reported the usage of an age-stage two-sex life table in elaborating the life history traits of *S. frugiperda* that were reared on different host plants (Guo et al., 2021; Xie et al., 2021) and the effects of spinetoram on its growth and fecundity (Gao et al., 2021). Similarly, several other studies were conducted to elaborate the impact of different insecticides on the life history traits of insects, such as *Spodoptera litura* (Rehan et al., 2015), *Oxycarenus hyalinipennis* (Lygaeidae; Hemiptera) (Ullah et al., 2016), and *Musca domestica* (Shah et al., 2015). Vitellogenesis is vital for the reproduction of oviparous insects since it includes the synthesis and absorption of vitellogenin (Vg), a source of nutrients for embryonic development as it stimulates oocyte maturation (Roy et al., 2018; Wu et al., 2020), which is required for reproduction (Schneider, 1996; Ge et al., 2019). Therefore, studying the difference between the two strains was crucial because insecticidal stress also affects fecundity.

Chlorantraniliprole, an anthranilic diamide, works by modulating ryanodine receptors (RyRs) in the sarcoplasmic reticulum membrane, causing permanent paralysis in insect bodies owing to acute muscular contraction caused by excessive Ca^2+^ release in the cytosol that causes insect feeding cessation and death (Nauen, 2006). It has been reported to eradicate various insect orders, including Coleoptera, Diptera, and Lepidoptera (Sattelle et al., 2008). As a new pesticide for managing lepidopterous pests, i.e., *Spodoptera exigua* and *Tryporyza incertulas*, it is considered a great alternative in integrated pest management (IPM) (Lai et al., 2011). However, prolonged use of chlorantraniliprole may impair the ecosystem; thus, its adverse effects on FAW must be evaluated to optimize the use and reduce environmental damage.

This study aimed to investigate the effects of chlorantraniliprole on the life history traits of susceptible and reduced susceptible strains of the field-collected population of *S. frugiperda* from Southwest China and the difference in vitellogenin content of the female strains. Moreover, the degree of chlorantraniliprole ferocity and its influence on all larval instars of *S. frugiperda* could appropriately highlight its efficacy. Therefore, the effects of chlorantraniliprole on the life stages (larva, pupa, and adult) of both male and female *S. frugiperda* could be used as a reference for its effective management under field conditions.

## 2 Materials and methods

### 2.1 Insects and insecticides

The field population of *S. frugiperda* was collected from Qianjiang (QJ-20), located in the southwestern part of China (N 29°32ˊ01, E 108°46ˊ15), during the summer of 2020. The bioassay result showed a moderate resistance with an RR of 10.39-fold against chlorantraniliprole. About 150–200 larvae were procured at the sample location. The larvae and adults were raised on synthetic food and 10% sugar syrup, respectively. Subsequently, we used sodium hypochlorite solution (0.2%–0.3%) to disinfect the fresh eggs and pupae. After the hatching of eggs, larvae at all growth stages were grown under a controlled environment at temperature, relative humidity (RH), and photoperiod of 26°C ± 1°C, 70%–80%, and 16:8 (light: dark), respectively. The target insecticide used for screening was 95% chlorantraniliprole (Corteva Agriscience, Indianapolis, United States).

### 2.2 Bioassays

The toxicity of chlorantraniliprole was determined in the third instar of *S. frugiperda* by topical application. At first, we determined the dose range for chlorantraniliprole in a preliminary experiment by using a series of its concentration (i.e., 200, 100, 50, and 10 mg a.i. L^−1^). Later, technical chlorantraniliprole was dissolved and diluted to a series of concentrations using acetone (i.e., ranged from 1.05 to 21.06 mg a.i. L^−1^) to determine the LD_50_ values of the subsequent generations of the two strains (established from the field population). A 50-μL micro-syringe, coupled with a micro-applicator (PB600-1 Repeating Dispenser, Hamilton Company), was used to apply 1 μL droplet of each prepared concentration on the dorsal side of the frontal thorax of the third-instar larva (average weight of 0.006 g) (Gutierrez-Moreno et al., 2019). Five different insecticidal concentrations and control (each in triplicate) were prepared, with 12 larvae in each replication. The LD_50_ values were expressed in µg g^−1^ (active ingredient/larval weight). A measure of 1 µL of acetone per larva was applied for the control treatment. The treated larvae were placed on a 12-compartment plate with enough food, with experiments performed in triplicate for each concentration. Mortality was observed at 48 h after treatment. The larvae that expressed severe inebriation signs (slow movement, twitching, feeding interruption, and severe growth inhibition) were considered dead.

### 2.3 Establishment of susceptible and reduced susceptible strains against chlorantraniliprole

Based on bioassay observations, the two strains derived from QJ-20 (RR = 10.39-fold) against chlorantraniliprole were prepared using the approach reported by Wang et al. (2006). About 200 F_6_-generation larvae of QJ-20 were divided equally into two groups: the susceptible strain (Crp-SUS G_12_) and the reduced susceptible strain (Crp-RES G_12_), using a single-pair mating method. The former generations were kept in the laboratory, without insecticidal contact, to free the field-collected population from biotic or abiotic stress.

For establishing the Crp-RES G_12_ strain, the screening doses, i.e., LD_70_ of each generation (6.31, 8.42, 9.47, 11.57, 12.62, and 13.68 mg a.i. L^−1^ from G_7_ to G_12_ generation, respectively) were prepared. About 250–300 third-instar larvae were treated topically with 1 μL droplet of each designed concentration on the dorsal side of the frontal thorax of the third-instar larva for each generation using a 50-μL micro-syringe (Hamilton Company, Reno, NV), coupled with a micro-applicator (PB600-1 Repeating Dispenser, Hamilton Company). After 48 h, the survived larvae were shifted into the glass tubes with a fresh artificial diet and raised for the next generation.

However, for the Crp-SUS G_12_ strain, male and female adults were coupled independently, and about 50 pairs were prepared during each generation. The progeny of about 20 pairs was considered for selection in every generation. A total of 20 third-instar larvae were given the same dosage of chlorantraniliprole (i.e., 5.26 mg a.i. L^−1^) in pair. Subsequently, after 48 h treatment, the mortality was checked, and the larvae with >80% mortality were raised for obtaining the next generation.

### 2.4 Life history traits

Based on the generational screening result of the two strains, a prominent resistance difference (i.e., 4.0-fold) was progressively developed. Then, about 100 adults of each strain were kept in a clean cage covered with a clean muslin cloth. At the time of high fecundity, we randomly selected at least five egg masses and allowed them to air-dry at room temperature. Later, 100 larvae from the hatched eggs were raised separately in an artificial diet in a pre-labeled glass tube with 1.8 cm diameter and 10 cm height. Larvae at all growth stages were monitored daily and transferred to a six-compartment dish/plate. All the eggs and pupae were disinfected using sodium hypochlorite solution.

The fresh adults (developed from artificially raised larvae) were paired, and each pair was grown separately in plastic cups (500 mL; 9.5 cm diameter and 13.7 cm height) to establish a family. Each cup contained a wet cotton ball soaked daily with 10% sugar solution. Eventually, 30 families of each strain were established, and the population characteristics, including longevity, fecundity, and developmental time, were recorded every day until the couple’s death. The eggs were precisely counted and recorded. The life parameters, including time and duration of each growth stage, the emergence, life span, mating, and fecundity intensity of the adults, were recorded. The dataset of the values was analyzed to establish the life table.

### 2.5 Protein contents of Vg and VgR

The ovaries from five adult female adults of each strain were collected 2 days after their emergence (Zhen et al., 2018), weighed. A measure of 1 mL of PBS (pH 7.3) was added and then manually homogenized. The supernatant was then collected by centrifuging the mixture at 2,500 g for 20 min. The protein contents of Vg and VgR were determined according to the instructions of the ELISA kit (Shanghai Enzyme Biotechnology Co., Ltd., Product Code: mlbio104703 for insect vitellogenin and mlbio104704 for insect vitellogenin receptor): Insect Vg (or VgR) was added to a microtiter plate well that had been coated with a particular insect Vg (or VgR) antibody and then combined with an antibody that had been labeled with horseradish peroxidase (HRP) to form an antibody–antigen–enzyme–antibody complex. After thoroughly cleaning the plate, 3, 3′5, 5′-tetramethyl benzidine (TMB) substrate solution was added until the substrate turned blue, signifying HRP enzyme catalysis. The reaction was then stopped by adding sulfuric acid solution. The concentrations of Vg (or VgR) were calculated by comparing the absorbance (OD) of the samples to the standard curve using a microplate reader (Model 680 Microplate Reader, Bio-Rad) that measured the absorbance (OD) at 450 nm.

### 2.6 Statistical analysis

The POLO 2.0 program (LeOra Software, www.leorasoftware.com) was used to calculate the slope, LD_50_, 95% fiducial limits (FLs), and chi-square (*X* ^*2*^) value of the insecticide after 48 h of treatment (Wang et al., 2018). The susceptible baseline value was referred from Wang et al. (2021). The raw data of the life table were examined using age-stage two-sex life table computer software. The basic parameters, including age-specific survival rate (*l*_*x*_), age-stage survival rate (*s*_*xj*_), finite rate of increase (*λ*), reproductive value (*v*_*xj*_), intrinsic rate of increase (*r*), net reproductive rate (*R*_*0*_), and mean generation time (*T*), were analyzed. Based on the confidence interval of the differences, the adult longevity, fecundity, adult pre-oviposition period (APOP), total pre-oviposition period (TPOP), developmental growth, and other population parameters (*r, λ*, *R*_*0*_, and T) were compared using the paired bootstrap test with 10,000 random resamplings. The intrinsic rate of increase (*r*) and the finite rate of increase (*λ*) are the two critical metrics for estimating the capacity for population expansion, used to indicate the population’s fitness. A sigma plot (SigmaPlot 12.0) was used for the graphical representation of survival rate and reproductive value curves.

## 3 Results

### 3.1 Screening of the Crp-RES G_12_ and Crp-SUS G_12_ strains from the field population

The third-instar larvae were screened for toxicity of the two strains: Crp-SUS G_12_ and Crp-RES G_12._ The increased toxicity to chlorantraniliprole was observed for Crp-SUS G_12_ (LD_50_: 0.349 μg·g^−1^), having almost similar susceptibility to the referred susceptible strain as observed by the overlapping FL (fiducial limit) of its LD_50_ values (Table 1). In contrast, the Crp-RES G_12_ strain showed reduced susceptibility to chlorantraniliprole (LD_50_: 1.63 μg·g^−1^), and the resistance ratio increased by four-fold compared to the susceptible strain. Moreover, no overlapping of CI for LD_50_ was observed between Crp-RES G_12_ and susceptible strains (Table 2).

**TABLE 1:** Dose–mortality response (µg/larva) for the chlorantraniliprole-susceptible strain.

Generation	Survival %	n^a^	Slope ± SE	LD_50_ (95% FL) (µg/g)	*X* ^2^ (df)	*p*	RR^b^
SUS*	—	—	1.139 ± 0.234	0.410 (0.229–0.602)	3.425 (18)	0.9990	1.00
Crp-SUS G_7_	67.85	216	3.088 ± 0.418	1.050 (0.868–1.302)	21.77 (16)	0.1507	2.56
Crp-SUS G_8_	59.12	216	3.074 ± 0.376	0.790 (0.675–0.918)	10.45 (16)	0.8420	1.93
Crp-SUS G_9_	50.00	216	2.761 ± 0.329	0.567 (0.470–0.669)	7.69 (16)	0.9575	1.38
Crp-SUS G_10_	39.35	216	2.460 ± 0.304	0.505 (0.409–0.606)	8.88 (16)	0.9182	1.23
Crp-SUS G_11_	35.19	216	2.701 ± 0.323	0.449 (0.365–0.534)	13.79 (16)	0.6143	1.09
Crp-SUS G_12_	28.24	216	2.705 ± 0.335	0.349 (0.276–0.421)	10.99 (16)	0.8101	0.85

Note: The parental generation selected for screening was G_6_, and the median lethal dose (LD_50_) was expressed as micrograms of active ingredient per larva. n^a^, number of larvae used in bioassay; SUS*, baseline referred to Wang et al. (2021); RR^b^, LD_50_ of the generation/LD_50_ of SUS.

**TABLE 2:** Dose–mortality response (µg/larva) for the chlorantraniliprole-reduced susceptible strain.

Generation	Survival %	n^a^	Slope ± SE	LD_50_ (95% FL) (µg/g)	*X* ^2^ (df)	*p*	RR^b^
SUS*	—	—	1.139 ± 0.234	0.410 (0.229–0.602)	3.425 (18)	0.9990	1.00
Crp-RES G_7_	57.94	216	3.864 ± 0.459	0.778 (0.676–0.882)	11.73 (16)	0.7623	1.89
Crp-RES G_8_	55.56	216	5.091 ± 0.638	1.112 (0.998–1.225)	4.08 (16)	0.9987	2.71
Crp-RES G_9_	60.32	216	4.812 ± 0.593	1.231 (1.068–1.413)	23.56 (16)	0.0995	3.0
Crp-RES G_10_	59.26	216	3.943 ± 0.525	1.409 (1.252–1.595)	5.09 (16)	0.9952	3.44
Crp-RES G_11_	63.89	216	4.409 ± 0.596	1.540 (1.382–1.737)	5.22 (16)	0.9945	3.76
Crp-RES G_12_	68.05	216	5.576 ± 0.752	1.643 (1.499–1.825)	10.85 (16)	0.8186	4.0

Note: The parental generation selected for screening was G_6_, and the median lethal dose (LD_50_) was expressed as micrograms of active ingredient per larva. n^a^, number of larvae used in bioassay; SUS*, baseline referred to Wang et al. (2021); RR^b^, LD_50_ of the generation/LD_50_ of SUS.

### 3.2 Developmental times and population parameters of various life stages of Crp-RES G_12_ and Crp-SUS G_12_ strains

The life history traits of both strains were significantly different from each other. The developmental times of varying life stages of Crp-SUS G_12_ were considerably longer than those of Crp-RES G_12_ (*p* < 0.05) (Table 3). The mean longevity of male and female adults, APOP (adult pre-oviposition period), and TPOP (total pre-oviposition period) for Crp-SUS G_12_ were significantly longer than those for the Crp-RES G_12_ (*p* < 0.05), while the fecundity of Crp-SUS G_12_ was considerably lower than that of Crp-RES G_12_ (*p* < 0.05) (Table 4). However, the mean generation time (*T*) of the Crp-SUS G_12_ strain was much longer than that of the Crp-RES G_12_ strain, but the intrinsic rate of increase (*r*), finite rate of increase (*λ*), and net reproduction rate (*R*_0_) of the Crp-SUS G_12_ strain were all significantly lower than those of the Crp-RES G_12_ strain (Table 5).

**TABLE 3:** Developmental time durations of various life stages of the Crp-RES G_12_ and Crp-SUS G_12_ strains.

Stages	Crp-RES G_12_		Crp-SUS G_12_	
	*n*	Mean ± SE (days)	*n*	Mean ± SE (days)
Egg	100	5.95 ± 0.03 b	100	6.0 ± 0.02 a
First instar	100	3.81 ± 0.05 b	100	4.58 ± 0.07 a
Second instar	99	3.94 ± 0.08 b	100	4.67 ± 0.09 a
Third instar	92	5.12 ± 0.08 b	100	6.18 ± 0.06 a
Fourth instar	85	5.6 ± 0.09 b	98	6.44 ± 0.06 a
Fifth instar	84	5.98 ± 0.09 b	96	5.24 ± 0.08 a
Sixth instar	82	6.01 ± 0.90 a	96	5.92 ± 0.12 b
Pupa	68	18.49 ± 0.27 b	66	22.94 ± 0.46 a
Adult	68	10.6 ± 0.28 b	66	14.82 ± 0.65 a

Means in the same row followed by different letters are significantly different (*p* < 0.05) using Tukey’s test in SPSS software.

**TABLE 4:** Adult longevity and female fecundity for the Crp-RES G_12_ and Crp-SUS G_12_ strains.

Parameter	Crp-RES G_12_		Crp-SUS G_12_	
	*n*	Mean ± SE (days)	*n*	Mean ± SE (days)
Total longevity (M)	34	65.65 ± 0.79 b	38	79.24 ± 1.24 a
Total longevity (F)	34	65.09 ± 0.62 b	28	73.43 ± 1.05 a
APOP	33	3.73 ± 0.20 b	27	4.04 ± 0.26 a
TPOP	33	58.21 ± 0.45 b	27	62.89 ± 0.71 a
Fecundity	34	414.26 ± 31.03 a	28	304.43 ± 21.92 b

The standard errors (SE) of the mean values were estimated using 10,000 bootstrap replications. Means in the same row followed by different letters are significantly different (*p* < 0.05) using Tukey’s test in SPSS software. APOP, adult pre-oviposition period; TPOP, total pre-oviposition period.

**TABLE 5:** Population parameters for the Crp-RES G_12_ and Crp-SUS G_12_ strains.

Parameter	Original		Bootstrap (mean ± SE)	
	Crp-RES G_12_	Crp-SUS G_12_	Crp-RES G_12_	Crp-SUS G_12_
*r*	0.08	0.07	0.082 ± 0.003 a	0.067 ± 0.003 b
*λ*	1.09	1.07	1.085 ± 0.003 a	1.069 ± 0.003 b
*R*_*0*_	140.85	85.24	140.86 ± 22.16 a	85.18 ± 14.92 b
*T*	60.47	65.69	60.47 ± 0.56 b	65.71 ± 0.79 a

The standard errors (SE) of the mean values were estimated using 10,000 bootstrap replications. Means in the same row followed by different letters are significantly different (*p* < 0.05) using Tukey’s test in SPSS software. *r*, intrinsic rate of increase (d^-1^); *λ*, finite rate of increase (d^-1^); *R*_0_, net reproductive rate (d^-1^); *T*, mean generation time.

### 3.3 Age-stage-specific survival rate (*s*_*xj*_) and reproductive value (*v*_*xj*_) of the Crp-RES G_12_ and Crp-SUS G_12_ strains

**FIGURE 1:**
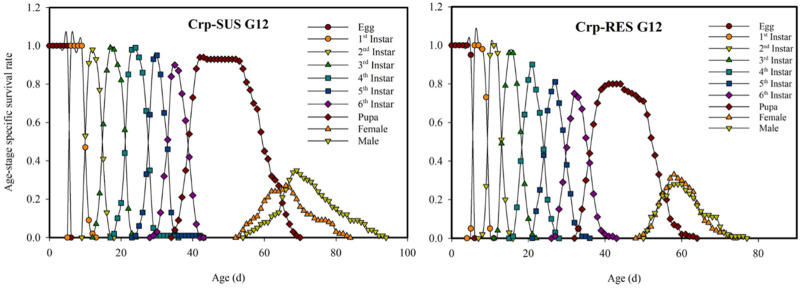
Age-stage-specific survival rate (*s*_*xj*_) for the Crp-RES G_12_ and Crp-SUS G_12_ strains of *Spodoptera frugiperda*.

**FIGURE 2:**
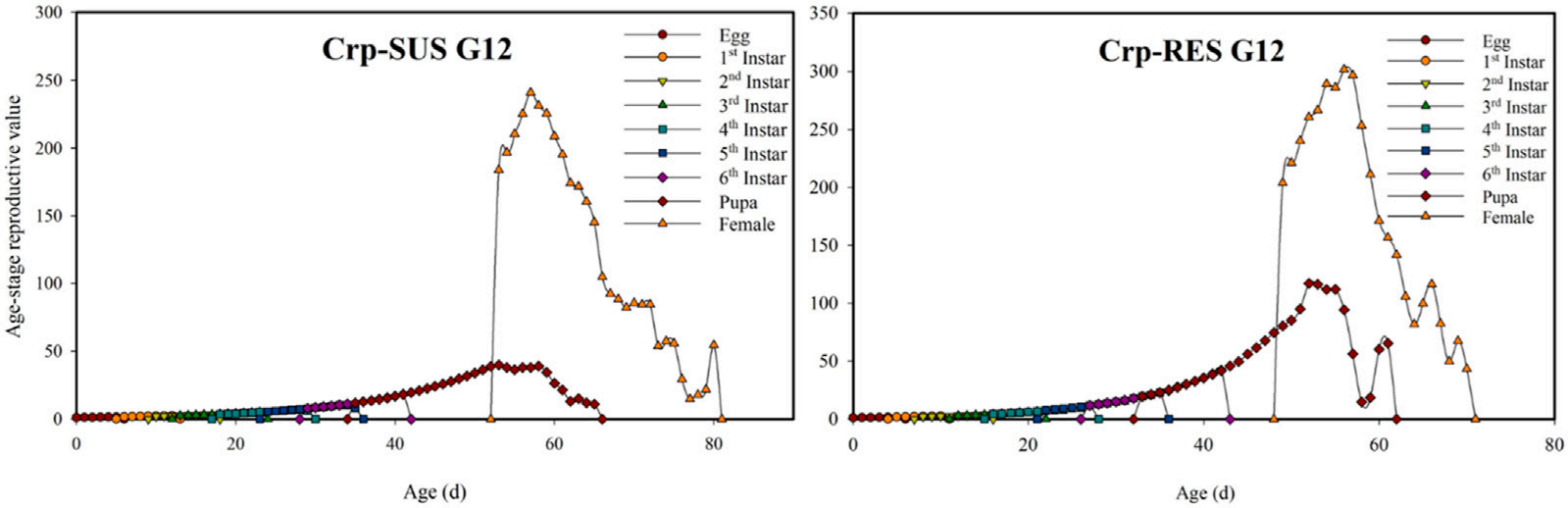
Age-stage reproductive value (*v*_*xj*_) for the Crp-RES G_12_ and Crp-SUS G_12_ strains of *S. frugiperda*.

The Crp-SUS G_12_ and Crp-RES G_12_ strains overlapped at various developmental phases and showed earlier terminating curves for female larvae than male larvae in the displayed peaks for each developmental stage. These peaks were more significant in the Crp-SUS G_12_ strain than in the Crp-RES G_12_ strain, indicating that male adults of the former had better survival rates. However, Crp-RES G_12_ female adults showed identical survival rates to male adults, but Crp-SUS G_12_ female larvae showed a lower peak and consequently poorer survival rates (Figure 1). During the pupal stage, the *v*_*xj*_ value of the Crp-SUS G_12_ individuals was lower than that of the Crp-RES G_12_ individuals. When the female adults emerged, the Crp-RES G_12_ strain’s plotted curve increased somewhat higher than the Crp-SUS G_12_ strain before significantly declining. In the Crp-SUS G_12_ strain, the maximum *v*_*xj*_ value was 241.1 d^−1^ on the 57th day, but in the Crp-RES G_12_ strain, it was 301.74 d^−1^ on the 56th day (Figure 2).

### 3.4 Age-stage-specific life expectancies (*e*_*xj*_) and age-specific survival rate (*l*_*x*_) of the Crp-RES G_12_ and Crp-SUS G_12_ strains

**FIGURE 3:**
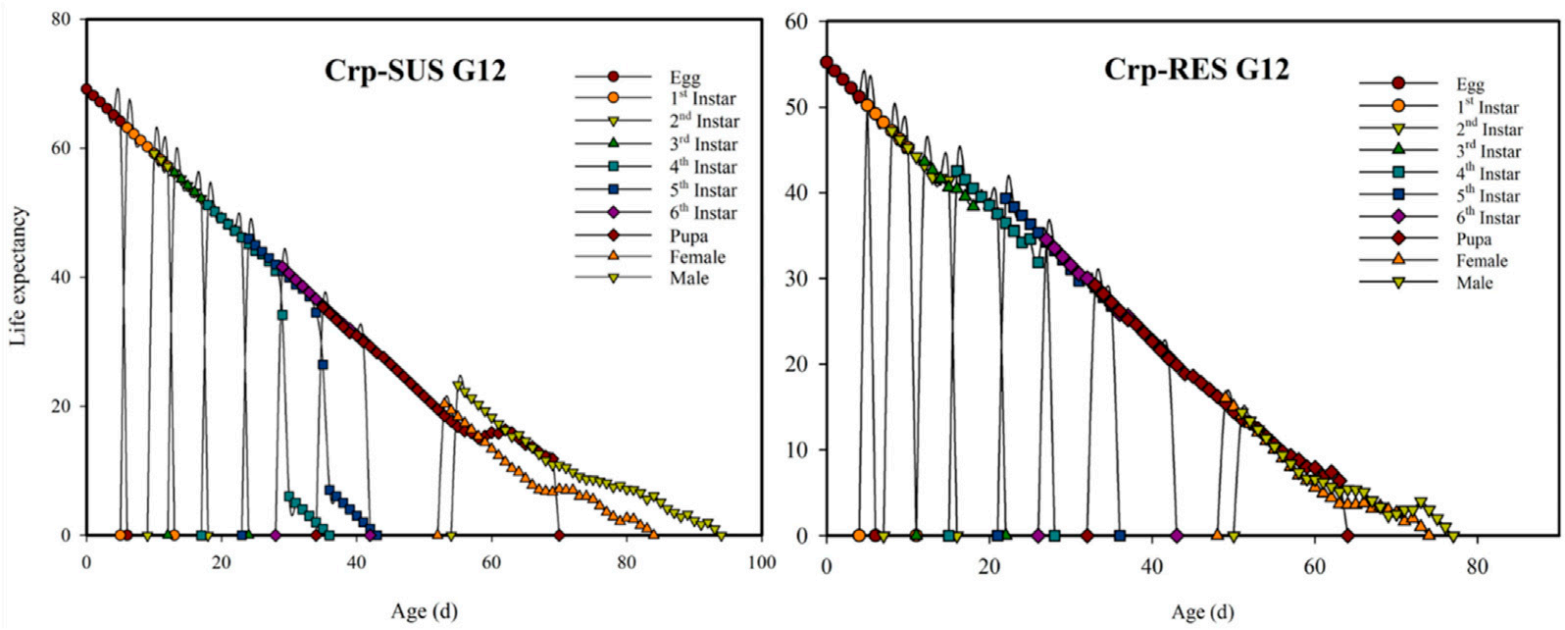
Age-stage-specific life expectancies (*e*_*xj*_) for the Crp-RES G_12_ and Crp-SUS G_12_ strains of *S. frugiperda*.

**FIGURE 4:**
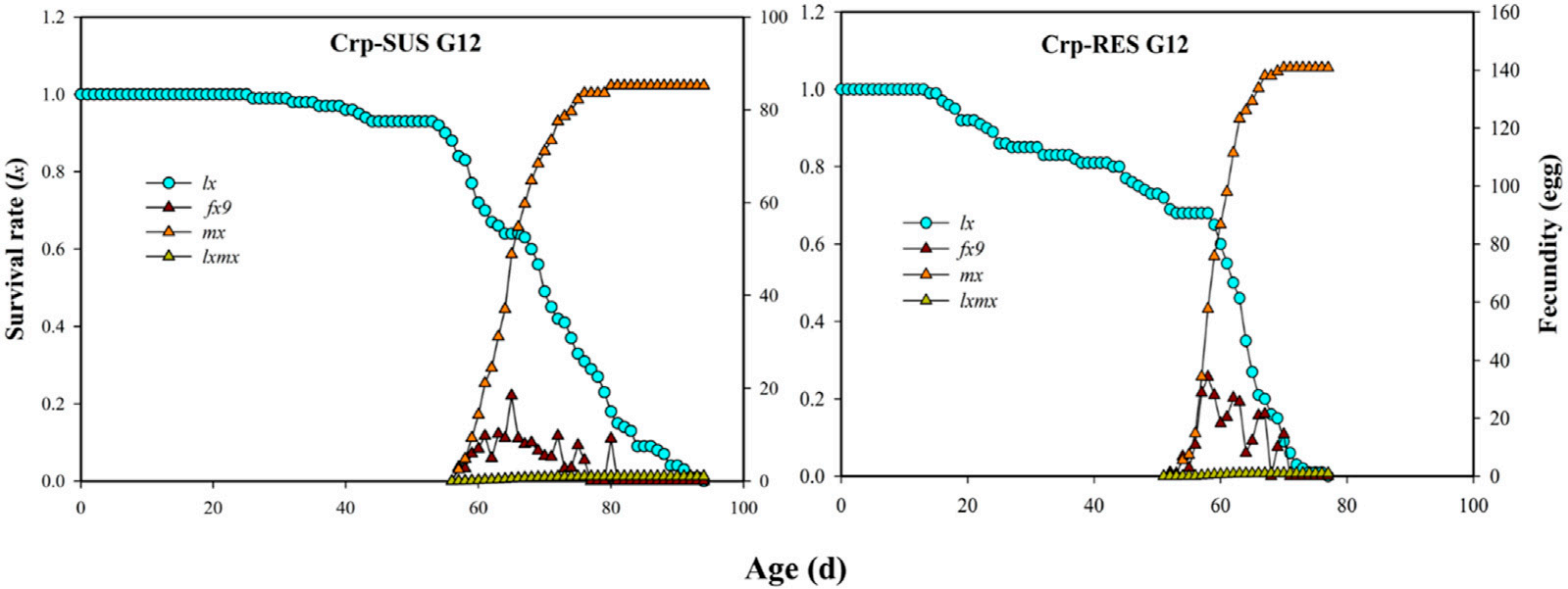
Age-specific survival rate (*l*_*x*_), female age-specific fecundity (*f*_*x9*_), age-specific fecundity of the total population (*m*_*x*_), and age-specific net maternity (*l*_*x*_ *m*_*x*_) of the Crp-RES G_12_ and Crp-SUS G_12_ strains of *S. frugiperda*.

In this research, the Crp-SUS G_12_ strain had a higher age-stage-specific life expectancy (*e*_*xj*_) than the Crp-RES G_12_ strain (Figure 3). The age-specific survival rates (*l*_*x*_) for these strains indicated that the *l*_*x*_ value decreased more quickly for the Crp-RES G_12_ strain at 15 d than the Crp-SUS G_12_ strain. In addition, the highest mean fecundity (*m*_*x*_) for Crp-RES G_12_ was 140.85 eggs compared to the Crp-SUS G_12_ that showed a mean fecundity of 85 eggs (Figure 4).

### 3.5 Contents of Vg and VgR in the Crp-RES G_12_ and Crp-SUS G_12_ strains

**FIGURE 5:**
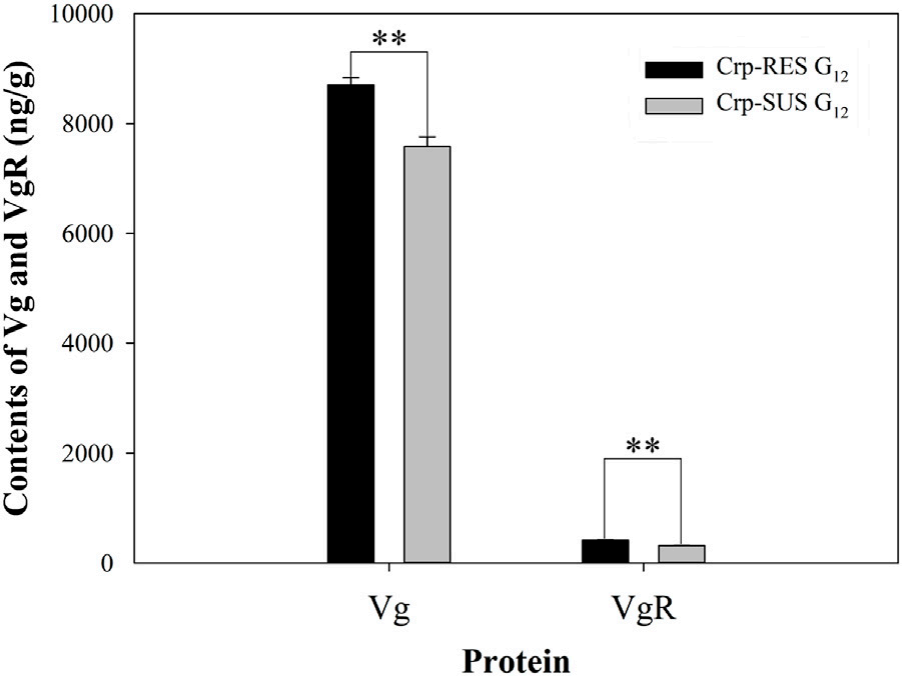
Contents of Vg and VgR for Crp-RES G_12_ and Crp-SUS G_12_ strains were presented as the mean of three replications ± SE. The asterisks above the bars indicate statistical differences between Vg and VgR contents of both strains (Student’s *t*-test, **p < 0.01).

The results indicated that the Vg content in the Crp-RES G_12_ strain (8,702.9 ng/g) was significantly more than that in the Crp-SUS G_12_ strain (7,581.7 ng/g) (*p* < 0.01). Similarly, the VgR content in Crp-RES G_12_ (414.6 ng/g) was significantly more than that in Crp-SUS G_12_ (318.8 ng/g) (*p* < 0.01) (Figure 5).

## 4 Discussion

The development of insect life tables is crucial for pest control methods when evaluating insect population dynamics (Gabre et al., 2005). Numerous biological and non-biological elements, including temperature, light, and the host plants, cause changes in insect populations (Tuan et al., 2014). Chemicals are currently considered one of the most effective integrated pest control strategies. However, the pervasive use of pesticides has led to substantial resistance, which is now a significant factor altering the parameters of insects’ life tables. Adequate knowledge about the life cycle, survival rate, and reproduction can be helpful in developing efficient management strategies against insect pests (Harcourt, 1969). In this study, we established the susceptible and reduced susceptible strains of *S. frugiperda* against chlorantraniliprole, and resistance ratios were gradually decreased (RR = 0.85 -fold) and increased (RR = 4.0 -fold) in both strains, respectively. Based on the results, all life stages of the Crp-SUS G_12_ strain had significantly longer time duration than those of the Crp-RES G_12_ strain, which is consistent with the findings of Abbas et al. (2012) in which imidacloprid application enhanced the emergence rate of healthy adults, developmental time, and fecundity of *S. litura*. In contrast, it was also reported that the fecundity of *S. litura* was reduced under the influence of emamectin benzoate, along with an increase in the larval duration and developmental time (Zaka et al., 2014), which was similar in the case of the Crp-SUS G_12_ strain in this study. Similarly, the thiamethoxam-selected individuals of *Mythimna separata* had prolonged larval and developmental times (Yasoob et al., 2018). This tendency suggests that different pesticides can alter the insect life history characteristics, which are also affected by many other variables, including the temperature (Chi et al., 2016), host plants (Nematikalkhoran et al., 2018), and insecticides (Steinbach et al., 2017).

Furthermore, chlorantraniliprole significantly affected the total pre-oviposition period (TPOP), mean longevities of male (M) and female (F) adults, and developmental times (T) of Crp-RES G_12_. These were positively related to the variations in intrinsic rate of increase (*r*), net reproductive rate (*R*), and finite rate of increase (*λ*). The fecundity and reproduction of both strains were greatly affected by chlorantraniliprole in this study. The plotted curves of the age-specific survival rate (*l*_*x*_) showed that the resistant strain showed the fitness cost of its normal survival rate and adapted to increase its eggs for better survival while shortening its life span. However, the Crp-SUS G_12_ strain modified itself toward only better survival and tried to maintain an average life duration. The age-stage reproductive value (*v*_*xj*_) also revealed the same trend with increased life span and decreased fecundity in the Crp-SUS G_12_ strain and *vice versa*. Similar results were also reported by Huang et al. (2019) that the bistrifluron-resistant strain of *S. litura* had higher values of population metrics along with an increase in fecundity than the bistrifluron-susceptible strain. However, in contrast, Han et al. (2012) showed that the fecundity decreased dramatically, along with the decrease in the values of *R, r*, and *λ*, when the sublethal doses of chlorantraniliprole were applied to the larvae of *Plutella xylostella*. Still, as in our study, we established Crp-RES G_12_ by screening LD_70_ of each generation (which is more than the median lethal dosage), and thus its effects are different from the sublethal doses. Moreover, the varied amounts of pesticides may contribute to differences between past and present results, based on the diverse species of insects evaluated and the pesticide application method.

Moreover, the fecundity in female adults is primarily governed by vitellogenin (Vg), and its receptor (VgR), as a precursor to Vg, is involved in the sequestration of Vg into the oocytes through endocytosis during the development and subsequently crucial for Vg uptake and oocyte maturation (Lu et al., 2015). The results of our study showed that the protein contents of Vg and VgR for Crp-RES G_12_ were higher than those for Crp-SUS G_12_, which supported the findings of Chen et al. (2020) that under the influence of triflumezopyrim, Vg and VgR contents increased in the subsequent F_4_ generation of *Sogatella furcifera*. In addition, Ge et al. (2011) reported that Vg content in the female larvae of *Nilaparvata lugens* significantly increased under the influence of triazophos. Similarly, Liu et al. (2016) also reported that the resistant strain of *Tetranychus cinnabarinus* treated with fenpropathrin had more protein content than the sensitive strain.

These results from life table data proved that the reduction in susceptibility against chlorantraniliprole positively affected the life history traits of *S. frugiperda*. However, to comprehend the impact of chlorantraniliprole on the life parameters of the individuals of Crp-SUS G_12_ and Crp-RES G_12_, further screening and follow-up research can be carried out to get more resistant individuals. In addition, as an alternate plan to integrated pest management, to prevent chlorantraniliprole from promoting the population dynamics of *S. frugiperda*, alternative no cross-resistance chemical insecticides or natural control agents should be used.

## 5 Conclusion

This study about the life history traits revealed that female adults of the reduced susceptible strain increased their fertility while lowering their life span to withstand chlorantraniliprole. In contrast, female adults of the susceptible strain decreased their fecundity while increasing their life span. These findings may serve as a foundation for subsequent research on chlorantraniliprole resistance in *S. frugiperda* and can contribute to developing the integrated pest management (IPM) program for fall armyworms in the future.

## Data Availability

The raw data supporting the conclusion of this article will be made available by the authors, without undue reservation.
