# Corrigendum: Effects of chlorantraniliprole on the life history traits of fall armyworm *Spodoptera frugiperda* (Lepidoptera: Noctuidae)

**DOI:** 10.3389/fphys.2023.1211689

**Published:** 2023-05-12

**Authors:** Ali Hasnain, Shuirong Zhang, Qinghua Chen, Lijuan Xia, Yutong Wu, Changwei Gong, Xuemei Liu, Pu Jian, Lei Zhang, Xuegui Wang

**Affiliations:** ^1^ State Key Laboratory of Crop Gene Exploration and Utilization in Southwest China, Sichuan Agricultural University, Chengdu, China; ^2^ College of Agriculture, Sichuan Agricultural University, Chengdu, China; ^3^ College of Plant Protection, Nanjing Agricultural University, Nanjing, China; ^4^ Key Laboratory of Integrated Pest Management on Crops in Southwest, Institute of Plant Protection, Sichuan Academy of Agricultural Sciences, Ministry of Agriculture, Chengdu, China; ^5^ Talent Development Service Center, Sichuan Provincial Department of Agriculture and Rural Affairs, Chengdu, China; ^6^ Department of Entomology, China Agricultural University, Beijing, China

**Keywords:** *Spodoptera frugiperda*, resistance, life history traits, chlorantraniliprole, vitellogenin

In the published article, there was an error in the **Acknowledgments** statement. The Acknowledgments statement should have been a **Funding** statement and the grant number “(22SYSX0138)” has further been corrected to “(2022YFSY0034).” The correct **Funding** statement appears below.

“The authors gratefully acknowledge the financial support of the Science and Technology Program of Sichuan, China (2022YFSY0034).”

The authors apologize for this error and state that this does not change the scientific conclusions of the article in any way. The original article has been updated.

